# LION Data: A roaring transformation in data visualisation

**DOI:** 10.1016/j.compchemeng.2025.109153

**Published:** 2025-05-14

**Authors:** Lucy Todd, Arthur Fordham, Ben Deacon, Marc-Olivier Coppens

**Affiliations:** a Centre for Nature Inspired Engineering & Department of Chemical Engineering, University College London, Torrington Place, London, WC1E 7JE, United Kingdom; b Electrochemical Innovation Lab & Department of Chemical Engineering, University College London, Torrington Place, London, WC1E 7JE, United Kingdom; c Chemistry and Chemical Engineering, University of Surrey, Stag Hill, University Campus, Guildford, GU2 7XH, United Kingdom

**Keywords:** Data visualisation, Scientific communication, Accessible software

## Abstract

In a world filled with high-quality scientific data, how much of it is truly being understood and utilised? LION Data is a Local, Interactive, Online, Networking data visualisation software specifically designed to address the requirements for scientists publishing data and users accessing it. This software allows Excel and CSV data to be efficiently uploaded through a ‘file browsing’ feature and then builds 2D/3D graphs and networks applying user requirements within seconds. This software has been shown to significantly improve the efficiency and breadth of data analyses conducted in a variety of scientific fields. Illustrative examples include 3D scaffolds designed to model biological environments, battery ultrasound readings to improve battery safety, and permeability readings of various chemicals on the skin. LION Data is easy to access, utilise, share and publish, allowing scientific data to be made accessible, understandable and available for any audience.

## 1. Introduction

Data is the core backbone of our web-based, technologically-driven society. What will the weather be like tomorrow? Which areas have affordable housing? What cars are most environmentally friendly? What is the best treatment available for a specific condition? These questions require a significant volume of research data to be answered confidently. The past 10 years have shown a notable increase in scientists publishing open-access research data (Tenopir et al., 2020; Borgman and Brand, 2024; Tedersoo et al., 2021). A study of 2000 scientists in 2017–2018 showed 85% of scientists would be willing to share their data if it were easy for others to access (Tenopir et al., 2020). This was a significant increase from a study carried out in 2003 where only 31% of scientists agreed they should share their data (Tenopir et al., 2011).

In addition to promoting collaboration among scientists, the increase in open-access publications has made this high-level, credible scientific data available to the general public (Cordasco et al., 2017). It has even been shown that papers with published data receive 25% more citations (Colavizzaid et al., 2020). Unfortunately, the format of such published data is often inaccessible or incomprehensible for the general public, and, sometimes, even other scientists (Cordasco et al., 2017; Hou and Mayernik, 2021). This has resulted in the FAIR principles (findable, accessible, interoperable, reusable) to promote more accessible and comprehensible data-sharing tools (Tenopir et al., 2020; Hou and Mayernik, 2021; Borgman and Brand, 2022). To accomplish these FAIR principles, internet-based, interactive, data visualisation dashboards have been proposed as a practical, easily adoptable tools for making data more discoverable, interpretable, and reusable (Hou and Mayernik, 2021; Rossi and Ahmed, 2016; Scheuer et al., 2021; Li, 2018).

The most common data analysis and visualisation tools in academia include Python, MatLab and RStudio. However, these require extensive coding experience that greatly increases the learning curve for non-coding users. There are various non-coding data visualisation tools currently available. However, many are highly specific (built for a single data type) (Shannon et al., 2003; Bartsch et al., 2014; Jones et al., 2016), still involve a significant learning curve (Bartsch et al., 2014), are inaccessible to non-scientific users (Shannon et al., 2003; Bartsch et al., 2014; Jones et al., 2016), require downloaded software (Bastian et al., 2009; OriginLab Corporation, 2023; Cloud Software Group Inc., 2025; JMP Statistical Discovery LLC., 2025), are expensive (OriginLab Corporation, 2023; JMP Statistical Discovery LLC., 2025; Cloud Software Group Inc., 2025), and are unable to locally or permanently house data (Cordasco et al., 2017; Catlin et al., 2018). Other data visualisation tools that specifically focus on building networks include Cytoscape.js (Shannon et al., 2003), Gephi (Bastian et al., 2009), Soc-NetV (Kalamaras, 2024) and GraphVis (Ellson et al., 2002). However, these packages mainly focus on showing pre-built networks (with edge–node connectivity determined) rather than constructing networks from adaptable, user-determined parameters.

To address this lack of comprehensive data visualisation software, we introduce LION Data (Todd, 2024). LION Data is a Local, Interactive, Online, Network dashboard specifically designed to address the requirements for scientists publishing their data and the users accessing it. The software is efficient, secure and easy to publish for scientists while being easy to learn, freely available, visually pleasing and interactive for the users. This software allows users to efficiently design and edit 2D and 3D graphs, which can be exported as PNG files. It builds interactive networks based on user inputs and inspired by Stuart Kauffman’s NK fitness landscape methodology (Kauffman, 1993). LION Data also provides filtered data tables that can be exported as Microsoft Excel or CSV files.

**Fig. 1.**
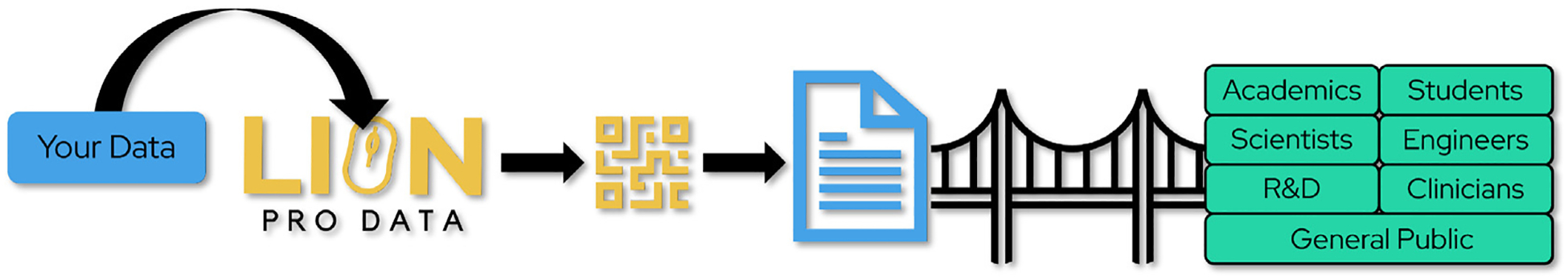
A schematic representation of how the LION Pro Data may be designed and attached to a published paper as a QR code, making the data more accessible and understandable to academics, students and the general public (represented by the bridge).

The HTML/Java Script LION Data version is freely available and provided in a Mendeley Data repository (the Python version is available upon request) (Todd, 2024). This dashboard can visualise up to 20,000 data points from any Microsoft Excel or CSV spreadsheet. There is also a Pro version available through the commercial branch of LION for a permanent, personalised, version of LION Data to house specific research data (information available upon request). This dashboard can then be hosted on a university’s, or journal’s website or attached to a publication increasing user engagement with the research data. A graphical description of LION Pro Data is shown in Fig. 1.

This paper includes the details of the four main features of LION that construct the acronym, Local, Interactive, Online and Networks. This is followed by a full description and schematic of the software including a comparison with Microsoft Excel and OriginPro (OriginLab Corporation, 2023). The paper concludes with three case studies demonstrating the versatility of the software to visualise and analyse data across a diverse array of fields including biomedical, battery safety and skin permeability. All software run times, unless otherwise specified, are conducted on a 32 GB, 11th Gen Intel(R) Dell laptop.

## 2. LION

### 2.1. Local

One of the main concerns for scientists publishing data or using data visualisation tools is security (Borgman and Brand, 2022). LION Data is designed to locally house all data. Any data used in the free, open-access version (provided in supplementary material, Todd, 2024) is processed locally on the user’s computer and may be completed without an internet connection. This ensures that all data remains within the user’s local environment. The Pro version of LION may be hosted on any server allowing scientists to choose where they want their data located, whether on a research lead’s group, a university’s, a company’s or a journal’s website. LION Data is written in both Python and HTML/Java Script, allowing clients to select the version beneficial for their specific application. This is a unique feature for LION Data, as it allows users to seamlessly integrate the software within their preferred HTML or Python framework. This adds an extra layer of accessibility relative to other data visualisation software, where a user has to learn bespoke software, which is time-consuming and expensive.

### 2.2. Interactive

**Fig. 2.**
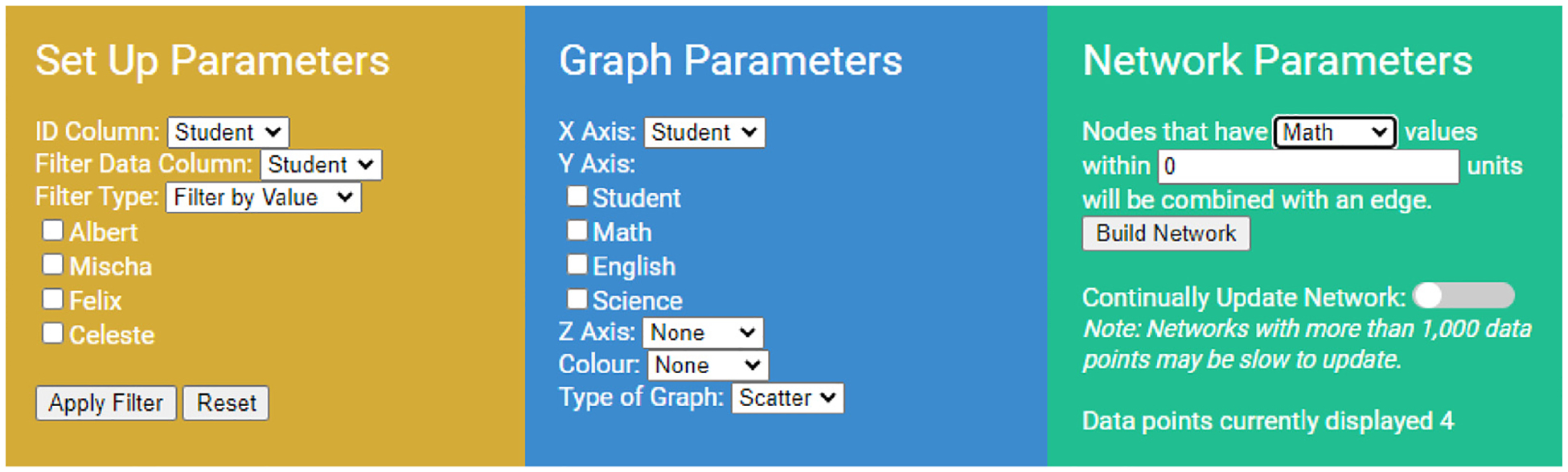
The three columns of drop-down menus in LION Data for general set up features, graph and network parameters. The image displays the parameters for a collection of students and their respective grades in various subjects.

Various studies show how dynamic, interactive data visualisation significantly increases usage compared to static visual displays (Rossi and Ahmed, 2016; Li, 2018). Therefore, LION Data contains columns of drop-down features that provide users with easily adaptable parameters shown in Fig. 2. These parameters include selecting the data used for the X, Y or Z-axis of the graph, whether the colour of the data points relates to data in the uploaded spreadsheet, the parameter used to build the networks, and various data filtering options. These drop-down features instantly update to contain the unique parameters from the Microsoft Excel or CSV spreadsheet uploaded by the user. The graphs and networks designed in LION Data are also interactive, allowing users to zoom in on graphs and networks, rotate 3D graphs, name the axes and titles, and click and drag nodes in the networks.

### 2.3. Online

LION Data is written in HTML/Java script, allowing users to open the software in their preferred internet browser without additional plugins or features. This may be completed with or without an internet connection, greatly enhancing accessibility by allowing users to select their preferred software hosting method without having to consider cost. This differs from Microsoft Excel and OriginPro (OriginLab Corporation, 2023), which require users to pay for the downloaded software and, in the case of OriginPro, does not offer an online platform.

This feature, along with the ease to learn the software, greatly increases user engagement (Hou and Mayernik, 2021). Studies examining users’ interactions with popular e-infrastructures (such as Earth System Grid Federation) (Chunpir et al., 2017) and students comparing large academic libraries with Wikipedia or Google (Matusiak, 2012) also demonstrate how the visual appearance of a web interface greatly impacts user engagement (Hou and Mayernik, 2021; Rossi and Ahmed, 2016; Li, 2018). Therefore, applying this information, LION Data is designed to have a stripped-back, clean, colourful and concise layout that allows users to quickly learn and utilise it (Chunpir et al., 2017; Hou and Mayernik, 2021).

### 2.4. Networks

**Fig. 3.**
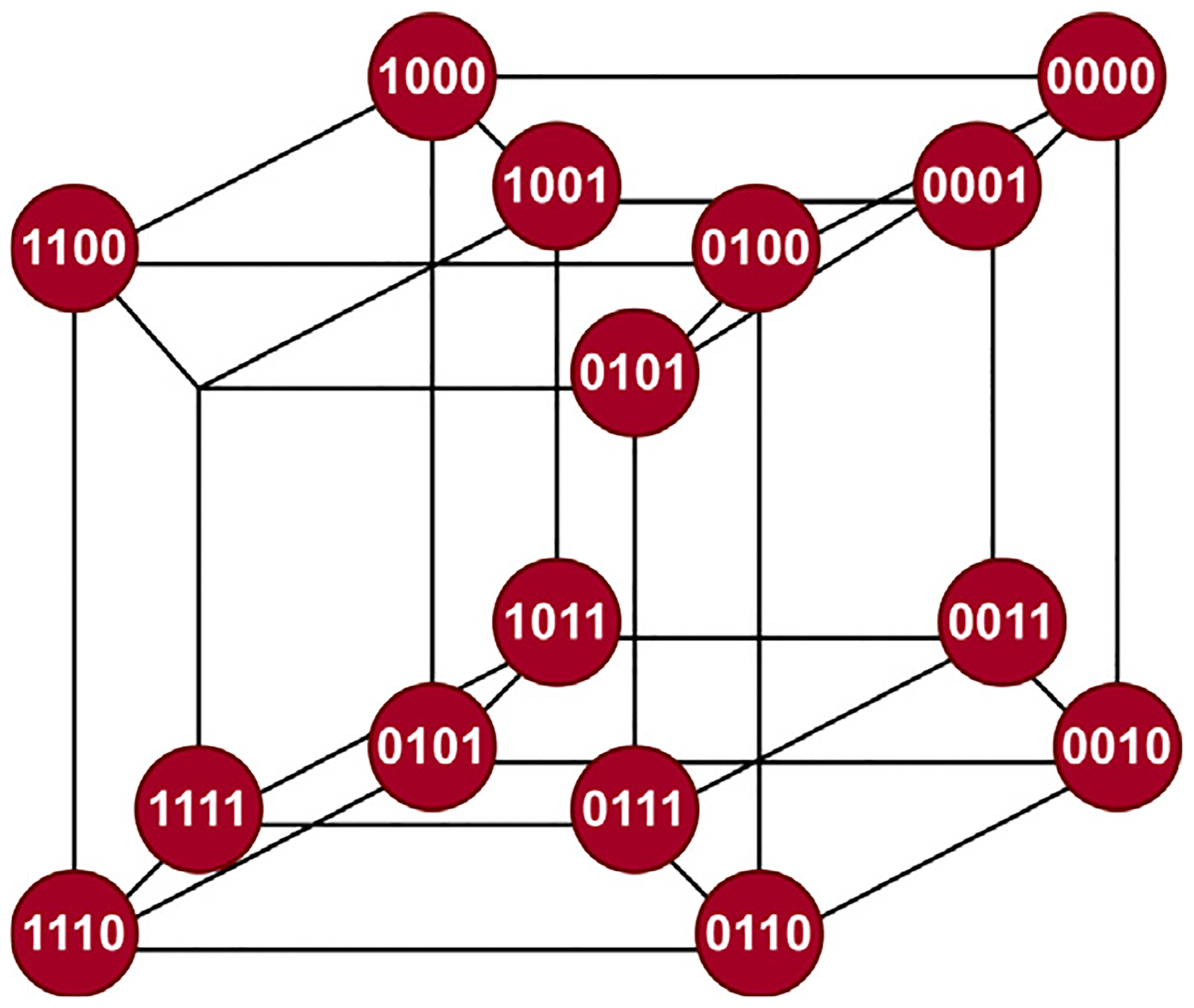
Kauffman’s NK fitness landscape for a simple *N* = 4 amino acid protein that consists of K = 2 amino acids (denoted by 0 or 1) where each vertex is a single configuration of this N-amino acid protein. The edges connect protein configurations that differ by just one amino acid (Kauffman, 1993).

In addition to designing 2D and 3D graphs, LION Data builds interactive and informative networks. These networks are constructed using the Nature Inspired Solution (NIS) methodology developed at the UCL Centre for Nature-Inspired Engineering (Coppens, 2021). The NIS methodology draws inspiration from nature to develop engineering solutions for global grand challenges. As part of this approach, various nature-inspired frameworks are examined, leading to the selection of Stuart Kauffman’s NK fitness landscape methodology for mapping simple protein evolution (Kauffman, 1993). Kauffman challenged the linear understanding of a protein evolving through single gene shifts by proposing a method to visualise all the possibilities of a single gene’s evolution (Kauffman, 1993). In Kauffman’s original NK fitness landscape, all the possible gene configurations were mapped for a four amino acid protein, composed of glycine and alanine. The different protein configurations were positioned as nodes, with edges connecting to other possible protein configurations that differ by just one amino acid (Fig. 3) (Kauffman, 1993).

**Fig. 4.**
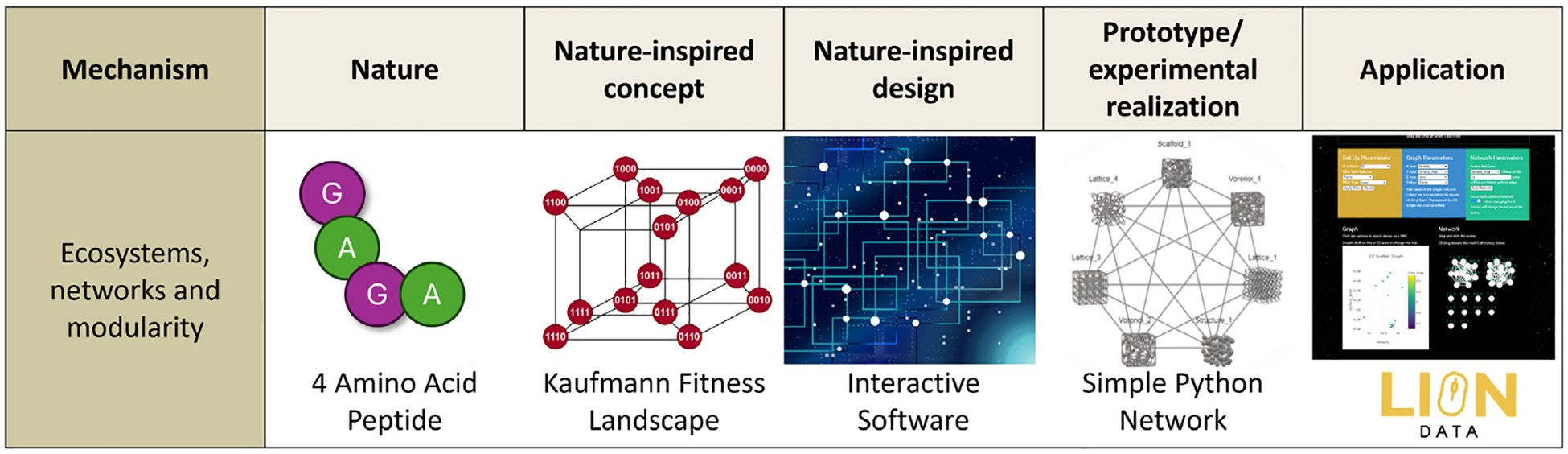
A visual schematic of the Nature Inspired Solution methodology (Coppens, 2021) applying Kauffman’s NK fitness landscape model (Kauffman, 1993) to this research.

Since Kauffman’s design in 1993, NK fitness landscapes have been adopted and developed in a wide variety of fields outside of evolutionary science including economics, management, anthropology, sociology, and law (Leprêtre et al., 2019; Gerrits and Marks, 2015). Over the years, NK fitness landscape networks have been extensively studied and refined, leading to the development of new theorems and improved analytical approaches (McCandlish, 2011; Sadler et al., 2023; Diao et al., 2023). Several specialised software platforms exist for modelling fitness landscapes, but these are typically constrained to evolutionary applications, require coding experience or are no longer readily available online (Brouillet et al., 2015; Padget et al., 2009; Huang and Li, 2023). Rather than replicating this extensive body of work, LION Data draws inspiration from Kauffman’s methodology to create an accessible software platform that extends the application of network-based analysis to a broader range of scientific data. To enhance efficiency, LION Data employs NK landscape principles tailored for general data exploration. A visual schematic of the NIS methodology applied to these networks is included in Fig. 4.

**Fig. 5.**
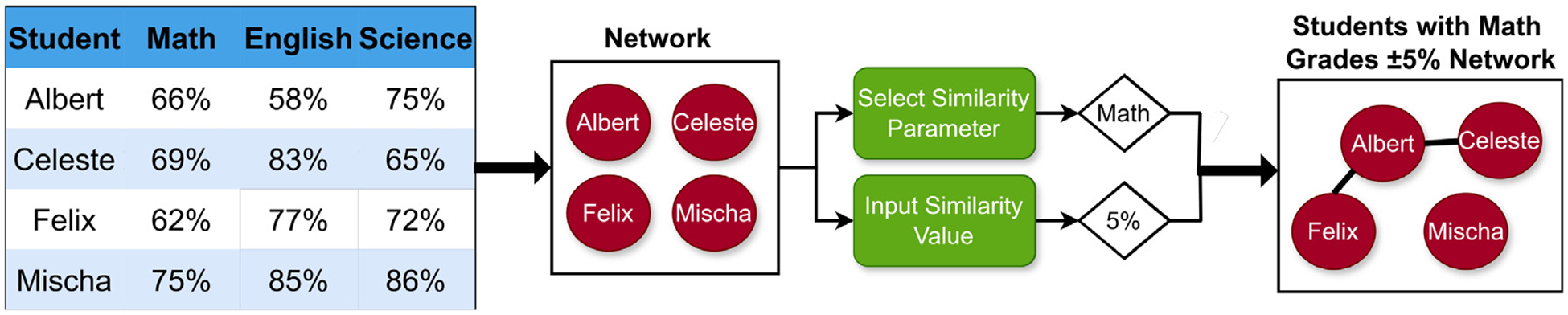
A schematic of how the LION Data software constructs networks. The data set of students and their respective grades in various subjects has been constructed for demonstration purposes only.

LION Data implements Kauffman’s methodology by designing networks based on the similarity of the various data points that the user has uploaded. Therefore, if a list of students and their final grades in various subjects is uploaded, a user may select any of these subjects (the Similarity Parameter - *ρ*) and build a network based on how similarly the students performed in that specific subject (the Similarity Value - *V*). For example, the user may select “Math” as the SP and then type “5” as the SV, resulting in a network where the various nodes (the students) are connected if they share a final Math grade within 5%. These nodes may be dragged around the screen and clicked to reveal the dictionary of data connected to that node. The process of how LION Data constructs networks is shown in Fig. 5.

To formalise this approach, the similarity between two nodes *i* and *j* is mathematically defined as,

(1) $$E\left(i,j\right)=\left\{\begin{array}{ll} 1, & \text{if}\;\left|\rho_{i}-\rho_{j}\right|\leq V\\ 0, & \text{otherwise} \end{array}\right.$$

where *E*(*i*, *j*) = 1 represents an edge between nodes *i* and *j*, *E*(*i*, *j*) = 0 represents no edge between nodes *i* and *j*, *ρ* is the selected similarity parameter, and *V* is the user-defined similarity value threshold. This formulation allows for efficient identification of relationships between data points, ensuring a dynamic and adaptable network representation. This structured approach ensures that even large datasets with thousands of entries can be processed and visualised in real-time, a significant advantage over traditional manual network-building methods.

## 3. Software design

**Fig. 6.**
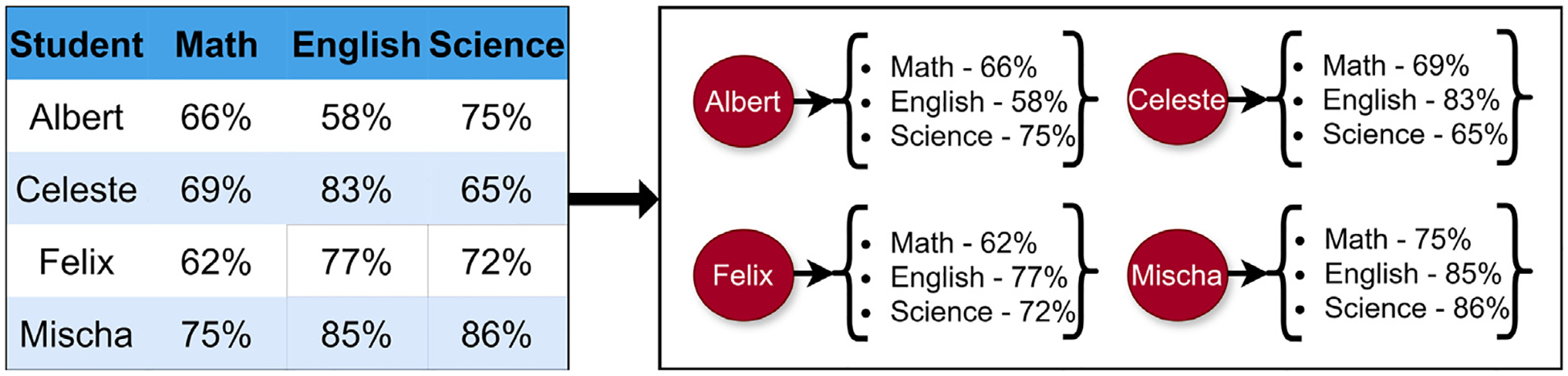
A schematic of the adjusting cycling algorithm in LION Data. The image on the left shows the initial input data frame (matrix *A*) and the image on the right shows the new node (*R*_1_ = Albert, *R*_2_ = Celeste, *R*_3_ = Felix, *R*_4_ = Mischa) and dictionary based system. In this example, *m* = 4 and *n* = 3.

The Python version of LION Data is built using the Dash-Cytoscape and Plotly packages (Plotly Technologies Inc., 2015). This code creates a website that takes multiple Microsoft Excel or CSV spreadsheets containing thousands of data points (rows - *m*) and their respective parameters (columns - *n*). Initially, the software would build graphs and tables in under 3 s but would crash when building networks with data sets even under 500 rows. This is because LION Data software was cycling through all the individual cells of the spreadsheet data frame separately *O*(*m* × *n*), placing a considerable demand on computational capacity and often causing the software to crash. This was addressed when the data frame was deconstructed and each row was redefined as a unique data point (a node) with its parameters listed within the node’s dictionary. The revised algorithm processes data row-wise instead of cell-wise, reducing the computational complexity to *O*(*m*). Mathematically, the time required for the previous iteration process was:

(2) $$T_{\text{old}}=\sum_{i=1}^{m}\sum_{j=1}^{n}f\left(A_{ij}\right),$$

where *A*_*ij*_ represents each cell in the spreadsheet data frame, requiring an operation on every cell; *f* is a function that determines the time it takes to perform Eq. (1); *T* is the total time. After optimisation, the time required for this process becomes:

(3) $$T_{\text{new}}=\sum_{i=1}^{m}f\left(R_{i}\right),$$

where *R*_*i*_ represents each row processed as a single entity, drastically reducing computational overhead. This redesign of the cycling algorithm significantly increases computational efficiency, allowing networks of 1000 rows to be built in under 10 s. This new design also efficiently converts back into a data frame to produce a downloadable Microsoft Excel or CSV file in under 2 s. This approach is shown in Fig. 6.

The HTML/JS version of LION Data is built using Plotly (Plotly Technologies Inc., 2015), allowing the software to be used locally or seamlessly integrated into any website. LION Data updates the interactive drop-down menus with the user-uploaded Microsoft Excel or CSV spreadsheet in under 2 s for spreadsheets up to 20,000 rows (which is the current limit). All further user adjustments result in instantaneous changes to the graphs and table. The networks may have a slight delay due to the complexity of defining the relationships between hundreds of rows of data. However, the delay is only between 5–10 s for up to 1000 rows of data. The delay in the network building is managed by providing users the option to build networks separately rather than updating automatically when the values in drop-down menus are adjusted. This feature can be turned off by flipping a toggle switch, as shown in Fig. 2.

**Fig. 7.**
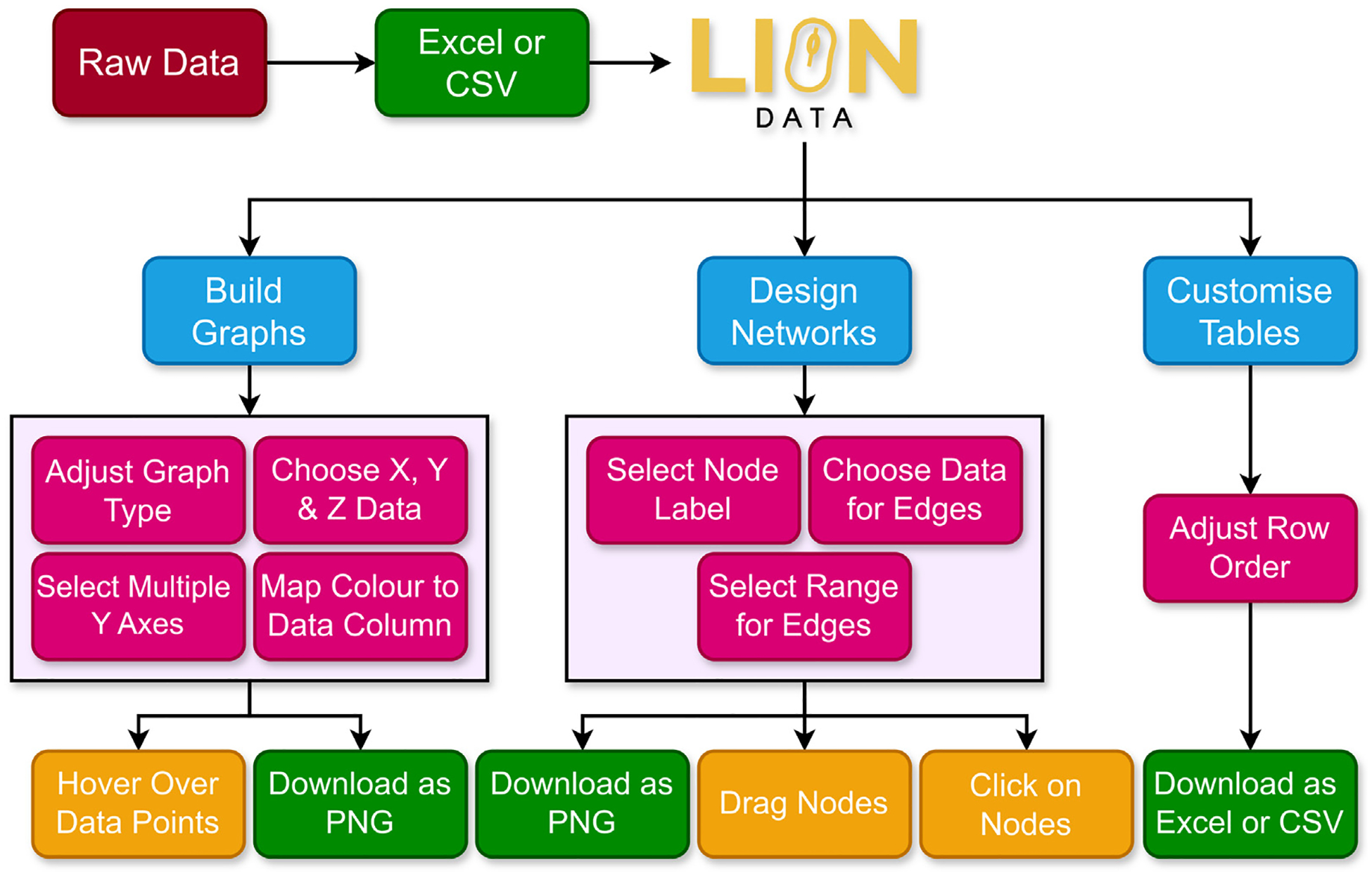
A full schematic of the LION Data software, which lists the data filter options (purple), three visualisation tools (blue) along with the various adjustable parameters (pink), user interactive features (orange) and downloadable options (green).

A full schematic of the data visualisation capabilities of LION Data is shown in Fig. 7.

### 3.1. Efficiency comparison

To compare the efficiency of LION Data against Microsoft Excel and OriginPro, seven data visualisation tasks are attempted and the number of mouse clicks (steps) required to complete each task is calculated. Various other comparison metrics, such as the time to complete the task, error rates and user experience, are also proposed. However, the number of clicks is chosen as the primary metric, because the other metrics are highly subjective to the abilities and experiences of a user with a specific software. The clicks result in instantaneous changes from the software (no lag time) and, therefore, provide an objective and informative measure of efficiency.

As shown in Table 1, LION Data is 5 times more efficient than Microsoft Excel in completing the first two visualisation tasks — the only tasks Excel can complete without individual programming. LION Data also performs marginally better than OriginPro when comparing the number of steps. However, the low number of steps in OriginPro was only achievable after multiple attempts and the realistic number was often closer to 20–35 clicks. Additionally, visualisation tasks performed in both Microsoft Excel and OriginPro required multiple internet searches for tutorials, with a few of these tasks only made possible through individual programming. Overall, LION Data proved to be more efficient and simpler when completing these tasks.

LION’s increased efficiency in analysing data, producing graphs and building networks (as shown in Table 1) is due to the streamlined and efficient layout of the software. However, this results in fewer user features such as customisable options, (font colour and size), the type of graphs, and statistical data analysis tools. Excel and OriginPro have a much wider array of features, aesthetic choices and data analytical options. However, the increased volume of options provided in Excel and OriginPro decreases user efficiency in producing more fundamental data visualisation (as shown in Table 1). Future versions of LION intend to incorporate further features available in conventional software while maintaining a lower learning curve and increased efficiency.

Additionally, the current version of LION prioritises ease of use, aiming to cater to users with a broad range of technical backgrounds, which may limit its appeal to those seeking the complex capabilities of more advanced software. We acknowledge that this trade-off may limit the depth of analysis required for more in-depth statistical tools or customisation options. However, our goal is to provide a versatile solution that allows scientists from diverse disciplines to visualise and interpret data quickly without the steep learning curve typical of other platforms. Future updates will address this gap by integrating more advanced functionalities while preserving LION’s user-centric design philosophy.

## 4. Case studies

The following three case studies demonstrate the wide applicability of LION Data within scientific research. Each study explores different areas of research and demonstrates how LION has been utilised to improve data processing and presentation.

### 4.1. 3D printed scaffolds

**Fig. 8.**
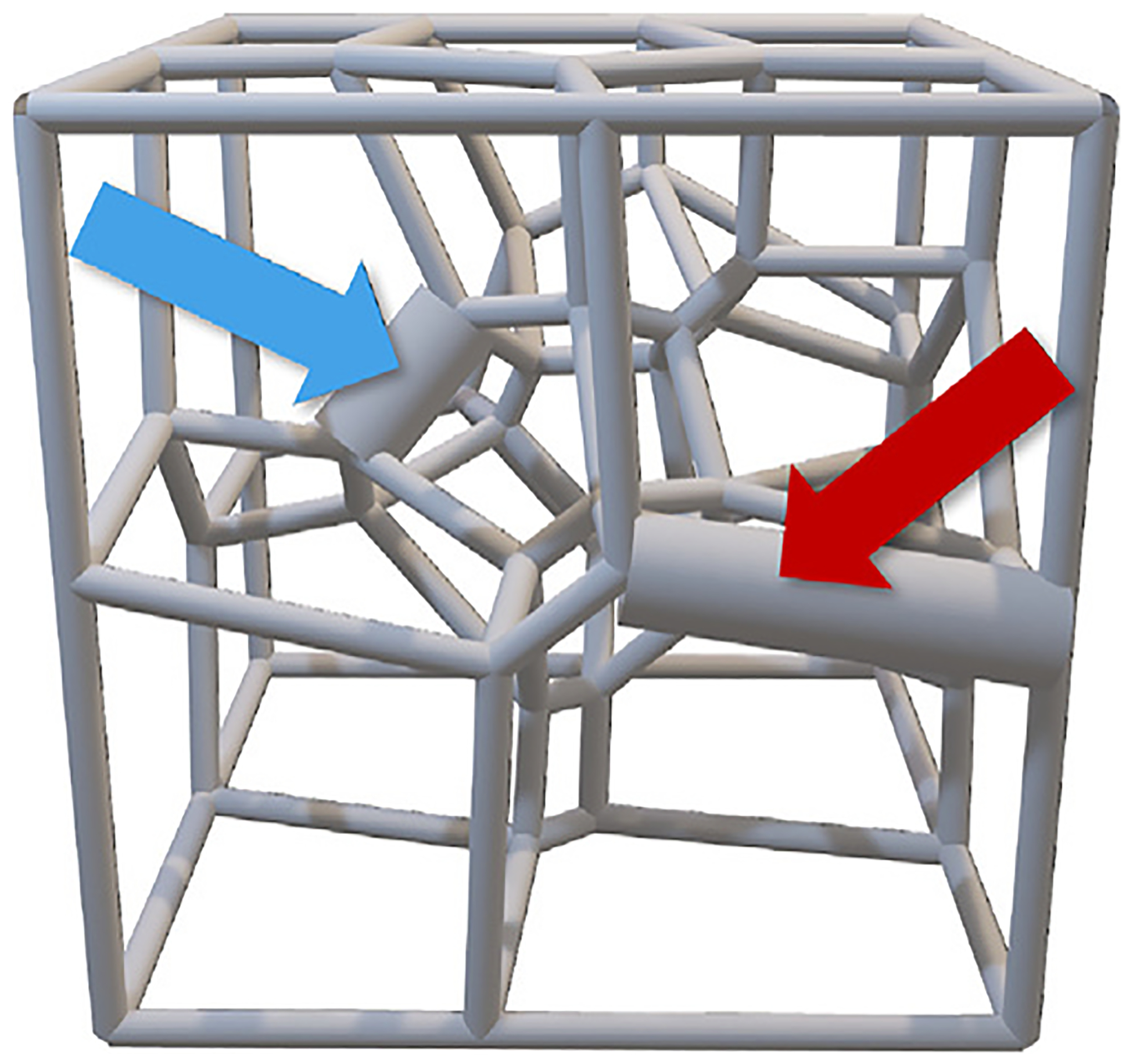
An example of a 3D porous structure designed to be 3D printed for various biomedical purposes (Todd et al., 2023, 2024). The minimum and maximum edge length have been enlarged and indicated with the blue and red arrow, respectively. These scaffolds are used to test the efficiency of LION Data.

The first case study is based on the research presented by Todd et al. in “Two conjectures on 3D Voronoi Structures: A Toolkit with Biomedical Case Studies” (Todd et al., 2023, 2024). For this research, 12,000 3D porous structures were designed to be 3D printed for various biomedical purposes, including improving cancer immunotherapy, constructing bone scaffolds and understanding tumour microenvironments (Fig. 8). Over 10 different parameters, such as porosity and surface area, are calculated for each scaffold. To efficiently and accurately analyse the thousands of scaffolds, the Microsoft Excel files holding this data are uploaded to LION Data. Using the graphing and networking features, multiple correlations are observed in under one hour. The process of analysing this volume of data had previously taken 3–5 days using Excel and individual Python scripts.

**Fig. 9.**
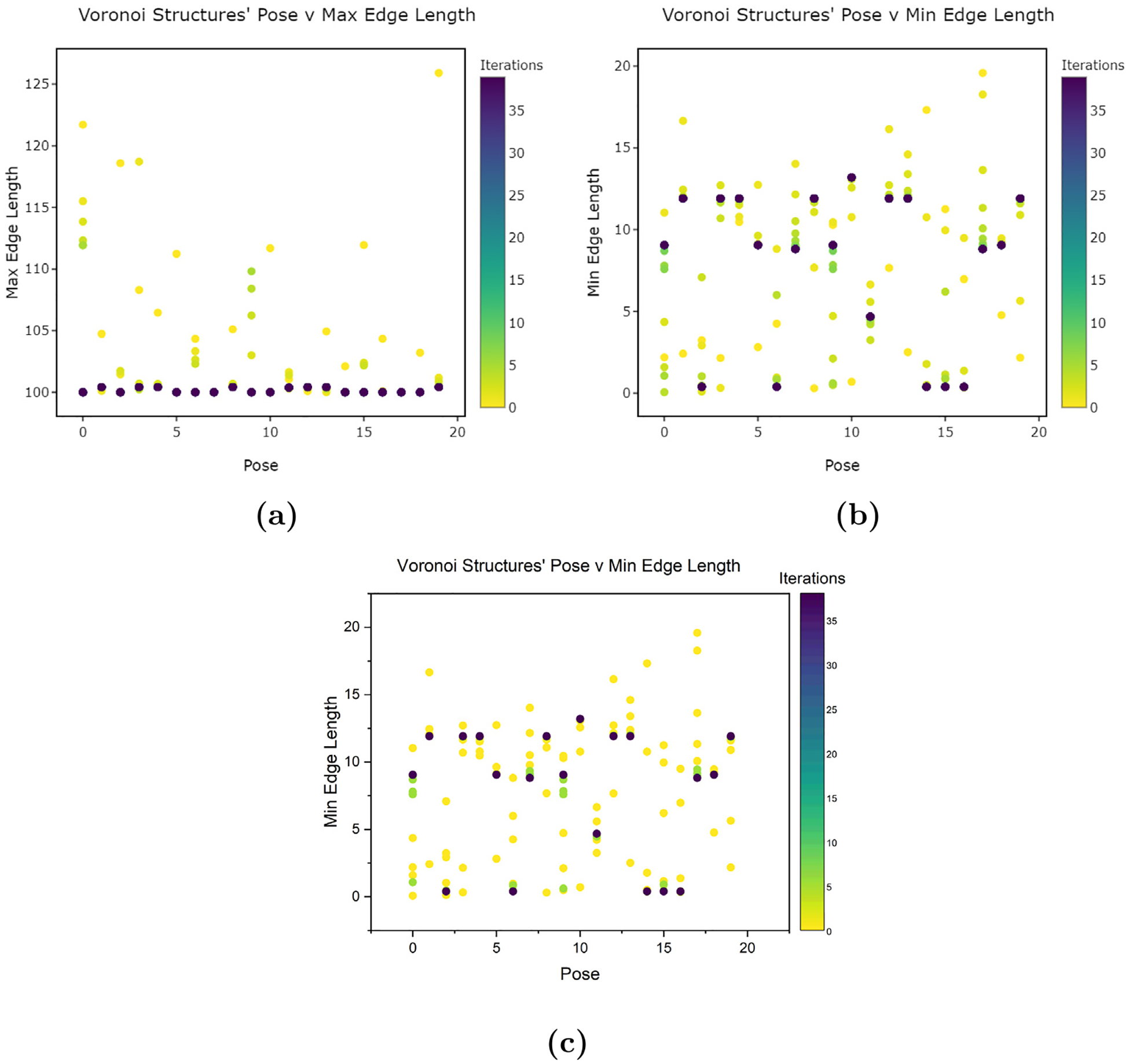
Various porous scaffolds grouped by their initial design feature (Poses) against their respective maximum (a) and minimum edge length (b,c). The colours indicate the number of geometric shifts each scaffold has gone through. Graphs (a) and (b) were built and exported through LION Data while (c) is through OriginPro.

Firstly, as the X, Y, Z axes and graph colours are adaptable instantaneously, every combination of the 10+ calculated scaffold parameters can be graphed efficiently. The scaffolds can be grouped based on their similar design features (Poses), with each scaffold going through a different number of geometric shifts that gradually adjust the lengths of their edges (Todd et al., 2024, 2023). Plotting the scaffolds against their respective maximum edge length, with the colour indicating the number of geometric iterations completed, shows a convergence towards a maximum edge length of 100 units as the number of geometric shifts increases (the purple data points shown in Fig. 9(a). This result is especially interesting as there is no convergence for the scaffolds’ minimum edge length, shown in Fig. 9(b). Using LION Data allowed these observations to be efficiently gleaned from the 10+ parameters within seconds. Further information regarding this research may be found within the original paper (Todd et al., 2024).

Secondly, it is possible to build and export these graphs within seconds. Although software such as OriginPro allows for extensive customisation options (such as font type and colour), OriginPro takes a long time to learn, requiring extensive tutorials to utilise it effectively, as discussed in Section 3.1. Graphs designed on OriginPro and LION Data reveal only minimal differences, as shown in Figs. 9(b) and 9(c).

**Fig. 10.**
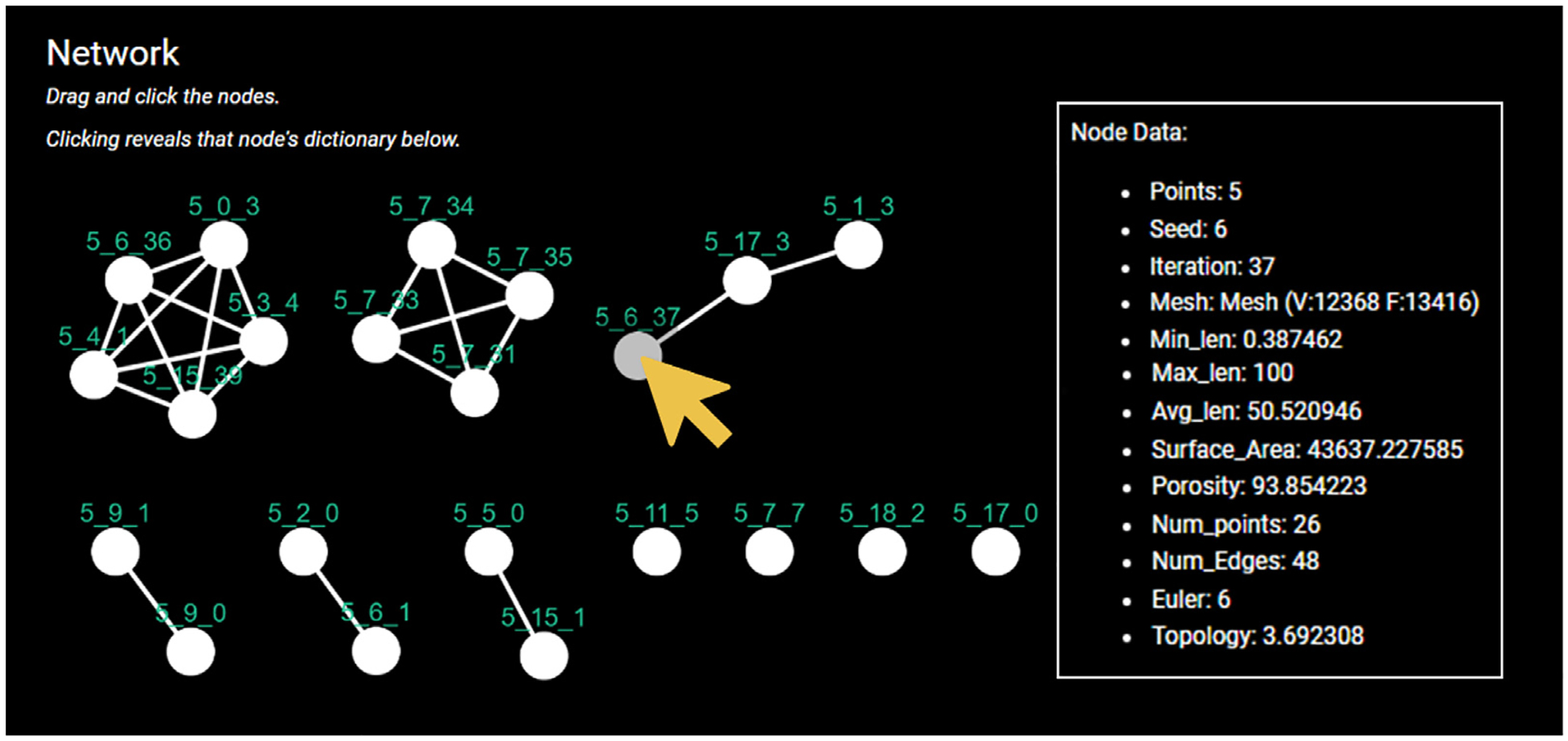
An example of a network built from LION Data. Each node is a 3D scaffolds connected to any others that share a surface area within 10 units. The user has “clicked” on the node labelled (as indicated by the arrow) which has resulted in that node’s dictionary of information being displayed in the box to the right called “Node Data”.

Thirdly, the network feature proves highly valuable in demonstrating which scaffolds hold the most distinctive or anomalous parameters. This network is important as only 2–3 out of 12,000 scaffolds will be 3D printed and the scaffolds with outlying parameter values are prioritised. As shown in Fig. 10, scaffolds with surface area values within 10 units are connected with an edge. This reveals which scaffolds hold similar surface area values reducing the number of prospective scaffolds to print, as only one from each cluster would be required, as representative for the cluster. It is then possible to examine the similarity of other features (such as porosity or maximum edge length) using only the scaffolds revealed to have unique surface area values through the LION Data filtering options. Through a series of iterations, it is possible to reduce thousands of scaffolds to 5–10 in under 10 min using LION Data. This task would have, previously, taken days or weeks as achieving this level of filtering would require users to write individual code in Python, MatLab or Excel.

### 4.2. Battery acoustic analysis

The second case study that utilised LION Data is based on research by Fordham et al. titled “Investigating the Performance and Safety of Li-ion Cylindrical Cells using Acoustic Emission and Machine Learning Analysis” (Fordham et al., 2024). Acoustic emission (AE) is a cost-effective and non-invasive diagnostic technique that employs a piezoelectric sensor to detect ultrasonic elastic waves generated by rapid energy release from a localised source (Kircheva et al., 2013; Etiemble et al., 2015; Yoshida et al., 2016). This method can assess battery safety and performance metrics across various battery formats, including cylindrical cells commonly used in electric vehicles (EVs) and consumer electronics. In their research, Fordham et al. correlated electrochemical performance with AE behaviour in cylindrical cells, studying differences during pristine and aged cell cycling. Over 5,000 AE data points were collected and used to train binary classifiers in a supervised setting, distinguishing between pristine and aged cells.

LION Data was vital for this project. Batteries are continuously producing AE events, especially when they degrade, causing an increase in cracks and movement within the internal structure. Each AE signal produced contains 14 parameters that characterise the signal (Rise Time, Counts, Energy, Duration, Amplitude (dB), Average Frequency, Root mean square (RMS), Average Signal Level, PCNTS (number of counts until the maximum amplitude is achieved), Threshold, Relative Frequency, Initial Frequency, Signal Strength, Absolute Energy, Time (min)). Over a period of 30 days of recording the acoustic signals produced from the cell, a substantial quantity of AE data points is amassed, each determined by these 14 parameters. Using former manual processing methods, such as individual Python scripts and OriginPro, this data analysis and plotting took at least a week to ensure clear trends and relationships were observed. However, by using LION, this same process took minutes.

In addition, due to the individualisation of the software’s design, a specific data “cleaning” backend is developed into the LION Data software used for this research. This allowed data produced from the ultrasonic AE technique to be directly uploaded into LION, where the data was formatted and organised correctly before data analysis and visualisation. The efficient nature of the software meant this could all be completed within a few seconds.

**Fig. 11.**
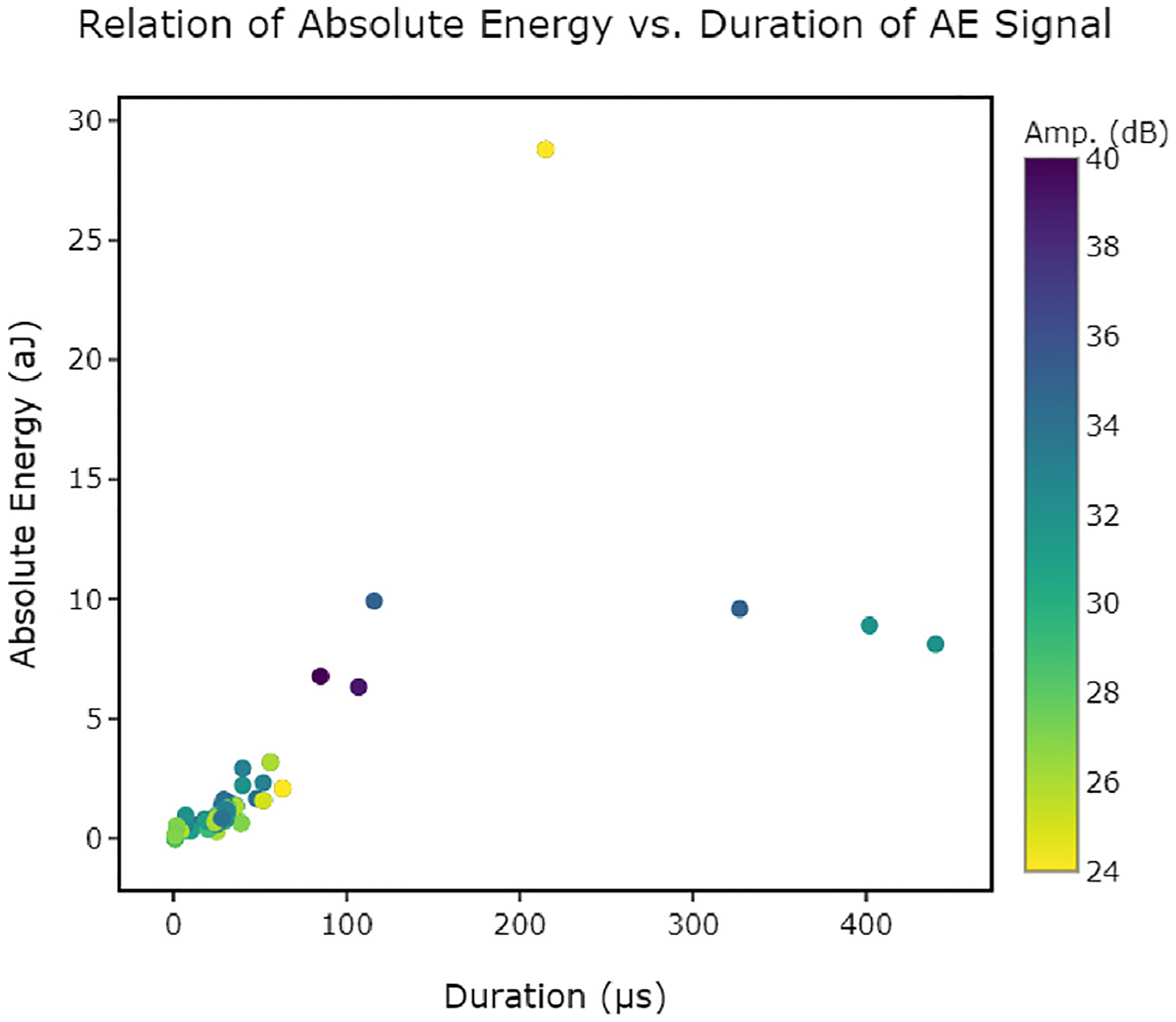
Trend observed for the relationship between Absolute Energy (aJ) and Duration (μs). Amplitude (dB) is represented by the colour bar (Amp.). Iterating through the different variables using LION data enabled this relationship to be discovered. The three outliers are illustrated in the plot.

Previously, Amplitude (dB) vs. Time (min) was the standard plot shown in AE literature (Majasan et al., 2021; Ramadan and Idrissi, 2008; Choe et al., 2015). While this relationship is important for understanding the type of signals released and their link to State of Health (SoH) of the battery, it does not provide a complete representation of the data. In contrast, LION Data’s processing methods ensured that it was easy and efficient to manipulate the data and plot each different permutation of the 14 parameters in 2D and 3D plots. The effectiveness of plotting each of these parameters and permutations against one another led to a far greater understanding of how AE can be used to understand battery degradation and improve its safety. Additionally, the ‘file browsing’ feature ensured that the different datasets could be added and processed simply and quickly. Links and relationships between parameters can be understood and visualised without the need for time consuming editing of code. For example, LION Data was used to plot ‘Absolute Energy vs. Duration (μs)’, revealing three clear outliers beyond a clear upward trend, (as shown in Fig. 11). Without LION, this relationship would not have been identified as efficiently, as each parameter was previously graphed separately in Excel.

**Fig. 12.**
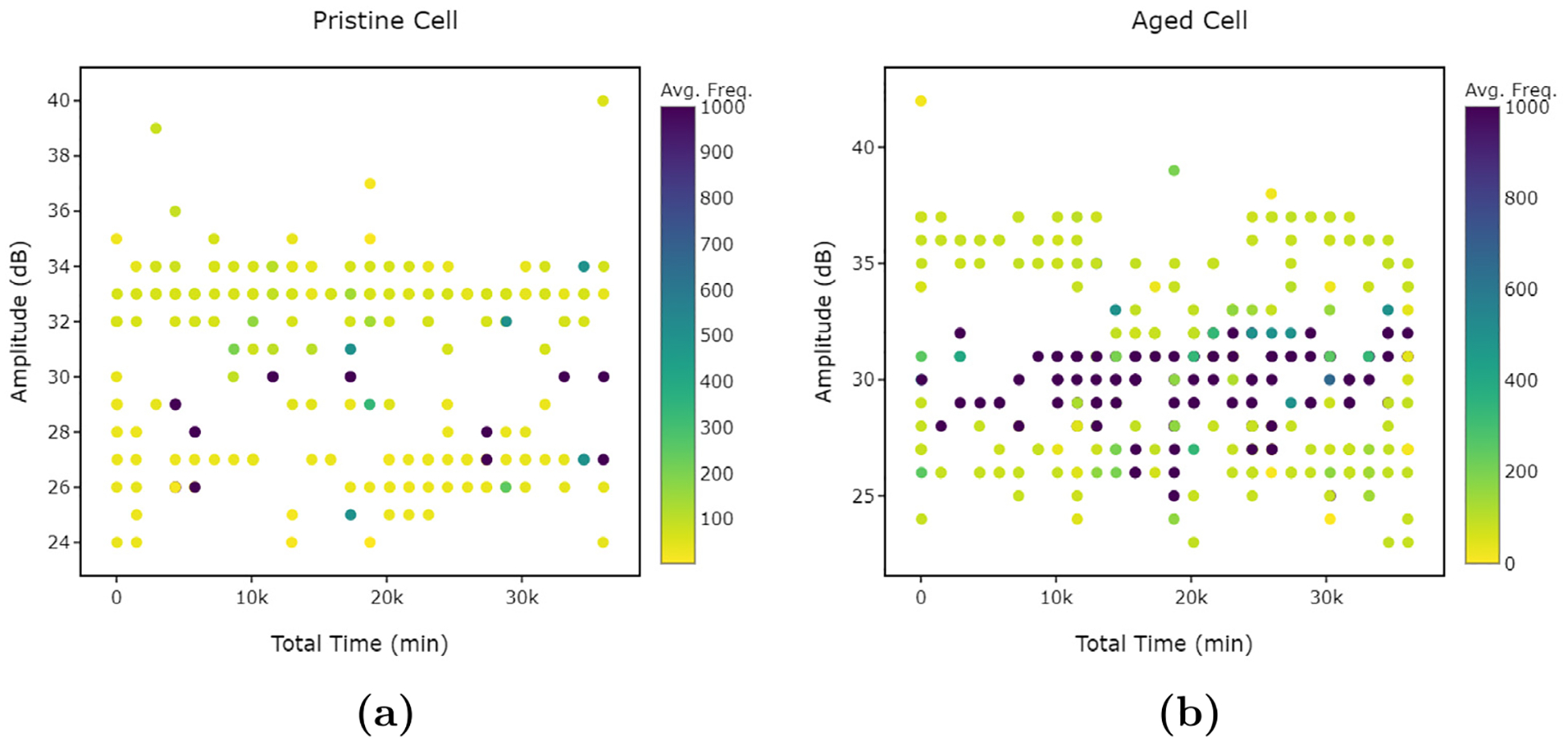
LION Data plots demonstrating how amplitude varies with time (min) and average frequency (avg. freq.) observed from a pristine (a) and aged (b) cell.

The colour scale, within LION Data, is useful to understand how the data are correlated. The colour scale allows for a clear distinction between the Pristine and Aged cells, with the Aged cell showing significantly darker signal colours in Fig. 12b compared to Fig. 12a. The plots demonstrate that the Average Frequency increases for the Aged cell (b) relative to the Pristine cell (a). Without LION’s colour scale feature, this distinction would be much harder to determine. Finally, Anomalous results can also be more easily identified using the different colours, which can then be related to the physical origin of the signal in terms of electrode cracking, gas formation, and electrode expansion.

Overall, LION Data ensured that each AE parameter could be related to another, and trends could be understood in relation to the physical origin of the signal from within the battery’s internal structure. The software provided key insights for this project on batteries, which now has the potential to be integrated into comprehensive cylindrical cell testing in both academic and commercial settings, aimed at enhancing the safety and performance of lithium ion batteries.

### 4.3. Permeability and dermal absorption

The third case study that used LION Data is from research by Deacon et al. titled “Computational Modelling of the Impact of Evaporation on *In Vitro* Dermal Absorption” (Deacon et al., 2024). Skin exposure to chemicals occurs regularly from the application of dermatological drugs and skincare products or unintended environmental pollutants (Anderson and Meade, 2014). Understanding dermal absorption and the impact of evaporation is paramount for the effective delivery of dermatological drugs and skincare products. *In silico*, physiologically based pharmacokinetic (PBPK) models are a supporting tool for more informed, faster, and cost-effective dermal absorption assessment (Chen et al., 2015). *In silico* models generate large datasets for each chemical assessed. For this case study the three datasets studied are PBPK-E (includes evaporation), PBPK and Hewitt (Hewitt et al., 2020), investigating 56 chemicals and calculating both the dermal delivery and permeability of each, resulting in 4200 data points.

Previously, Microsoft Excel had been used to visualise the data. However, plotting is slow for large datasets and can become confusing when trying to plot multiple datasets or making changes to existing plots. LION Data is significantly more efficient in visualising data and revealed two major trends. When comparing LION data and Excel on a local machine (2.3 GHz 8-Core Intel Core i9) the user has found that Excel requires 3 min of processing time to plot and apply visual changes to graphs containing 8400 data points, for 56 chemicals plotting the time course data in each skin layer. LION Data loads and plots this in a third of the time.

**Fig. 13.**
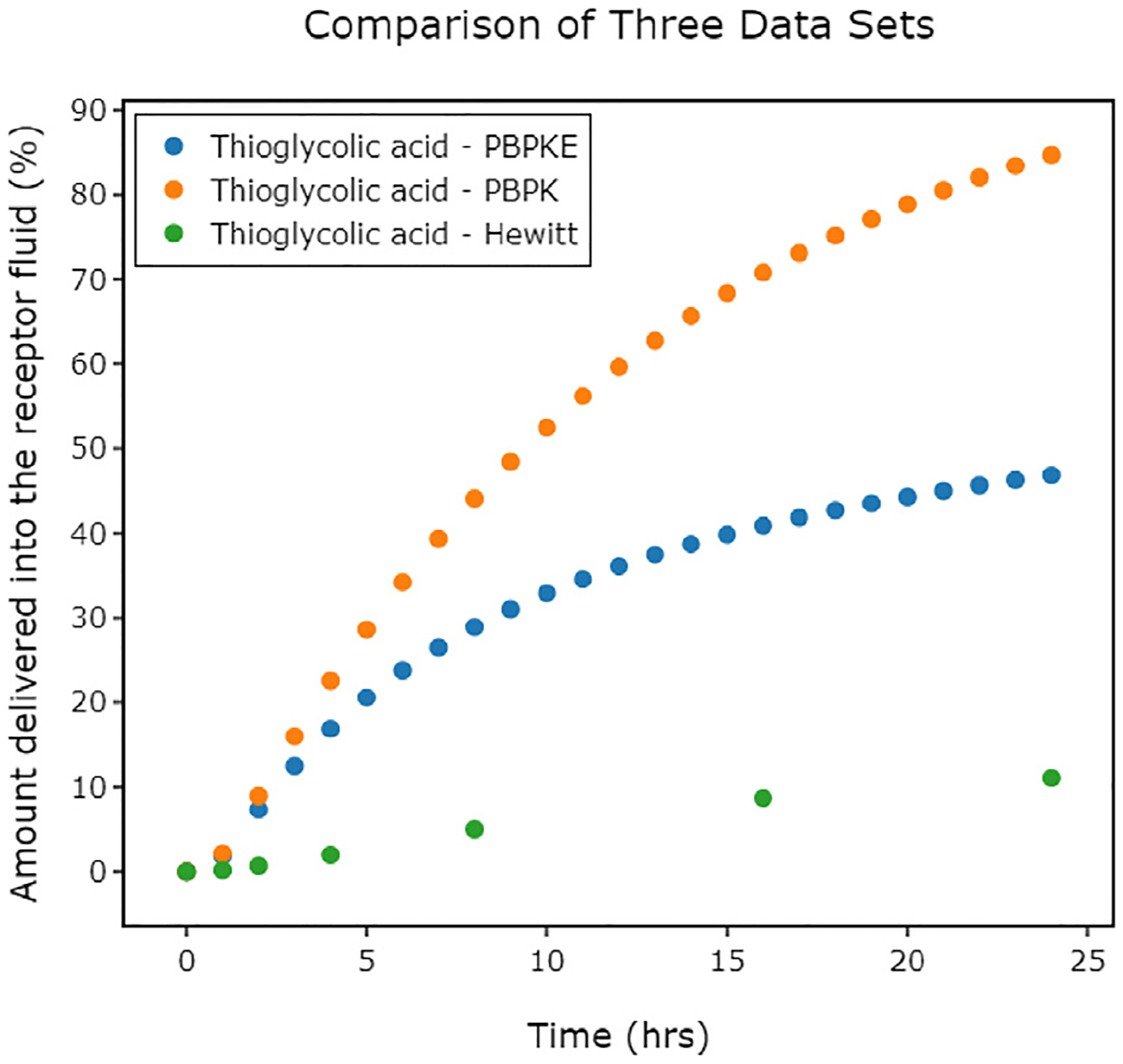
A LION Data 2D graph showing the percentage of thioglycolic acid delivered into the receptor fluid (RF) in three datasets: PBPK, PBPK-E and Hewitt (Hewitt et al., 2020).

Firstly, as LION Data allows for multiple datasets to be visualised on a single graph, the same chemical from each of the three datasets listed above (thioglycolic acid) is plotted on a single graph (Fig. 13). This clearly shows the impact evaporation has on the receptor fluid delivery between the different datasets.

**Fig. 14.**
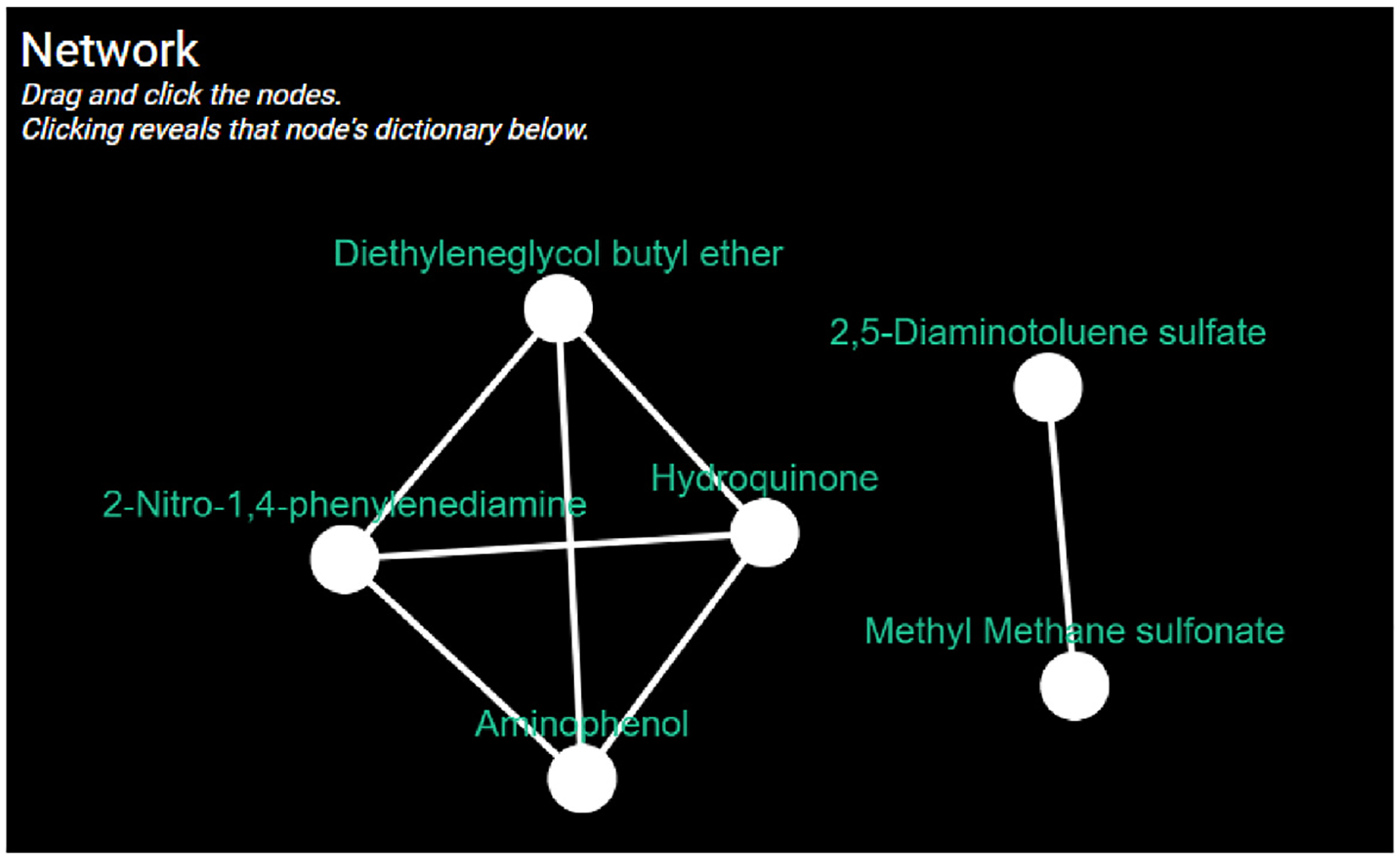
A LION Data network connecting a selection of chemicals that share Log ***K***_*ow*_ within 0.1 units.

Secondly, LION Data’s ability to create a network allows the chemicals to be grouped by physicochemical properties and analysed accordingly. For example, the network can highlight chemicals that may have a similar permeability or kinetic behaviour due to closely related properties. This can speed up the screening process to find chemicals with similar behaviour for screening novel drugs or potential active ingredients in formulations. A group of chemicals is shown with log ***K***_*ow*_ within 0.1 units (shown in Fig. 14).

**Fig. 15.**
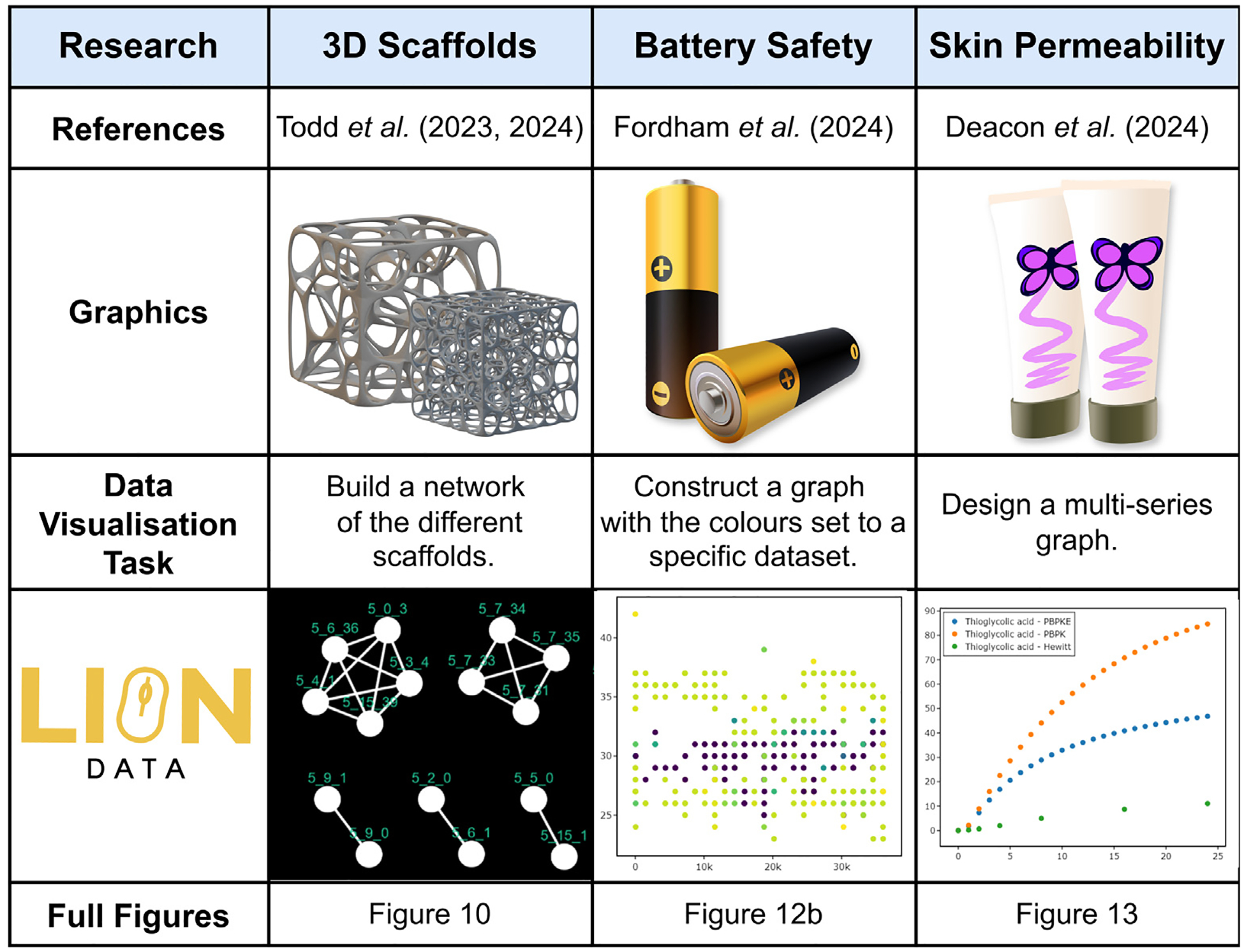
A summary of three case studies demonstrating a selection of data visualisation tasks completed in LION Data.

Finally, LION Data provides a standard reporting method making the results easier to read and understand. Another benefit seen in this work is the ability to disseminate data to others. Collaborators can now access the visualised data without having to plot their own graphs or requiring the author to generate 56 graphs to show each chemical’s behaviour (as demonstrated in Fig. 1). Furthermore, the use of LION Data removes the learning curve associated with other software, such as Python, Plotly and MatLab. Although they can provide similar benefits, such as a robust 3D networking plotting function, the user is required to code which is less common in experimental pharmaceutical groups. This software provides a convenient and efficient method for analysing and sharing data. A summary of these three case studies is shown in Fig. 15.

## 5. Conclusion

LION Data is a Local, Interactive, Online, Network dashboard specifically designed to address requirements for scientists publishing data and the users accessing it. This software has been shown to significantly improve the efficiency of data analyses, reducing days and weeks worth of analyses to minutes or hours. While LION Data prioritises accessibility, its backend design ensures computational efficiency by streamlining data handling, reducing unnecessary processing steps, and improving response times for large datasets. The benefits of this software have been demonstrated in research topics as versatile as 3D scaffolds designed to model biological environments, battery ultrasound readings to improve battery safety, and permeability readings of various chemicals on the skin. However, as LION Data may be used for any Microsoft Excel to CSV file, the potential applications reach far beyond the studies discussed in this paper. LION Data is easy to access, utilise, share and publish allowing scientific data to be made accessible, understandable and available for any audience.

## Figures and Tables

**Table 1:** A comparison of the features and efficiency of LION Data, Microsoft Excel and Origin Pro. Realistically, completing the task required multiple attempts and internet searches.

Visualisation task	LION	Microsoft Excel	OriginPro
Build 2D graphs	3 clicks	15+ Clicks	7 clicks
Build multi Y axis 2D graph	4 clicks	20+ Clicks^b^	7 clicks^b^
Build 2D graph with colour map	4 clicks	N/A^a^	15+ Clicks^b^
Build 3D scatter graph	4 clicks	N/A^a^	7 clicks^b^
Rotate 3D graph	Yes	N/A	Yes
Automatically update axis labels	Yes	No	Yes
Create networks	Yes	No	No

a Features that would only be possible with individual programming.

b This represents the minimal number of steps.

## Data Availability

The data have been shared as a Mendeley Data Repository.
